# Bufei Huoxue Capsule Attenuates PM2.5-Induced Pulmonary Inflammation in Mice

**DOI:** 10.1155/2017/1575793

**Published:** 2017-02-27

**Authors:** Yue Jing, Hongchun Zhang, Zhe Cai, Yukun Zhao, Ye Wu, Xuan Zheng, Ying Liu, Yuying Qin, Mingjie Gu, Jin Jin

**Affiliations:** ^1^Graduate School, Beijing University of Chinese Medicine, 11 North 3rd Ring East Road, Chaoyang District, Beijing 100029, China; ^2^National Clinical Research Center for Respiratory Diseases and Traditional Chinese Medicine Department of Pulmonary Diseases, China-Japan Friendship Hospital, 2 Yinghua Dongjie, Hepingli, Chaoyang District, Beijing 100029, China; ^3^Clinical Research Institute, China-Japan Friendship Hospital, 2 Yinghua Dongjie, Hepingli, Chaoyang District, Beijing 100029, China; ^4^Institute of Basic Theory for Chinese Medicine, China Academy of Chinese Medical Science, 16 Dongzhimennei Nanxiaojie, Dongcheng District, Beijing 100700, China; ^5^School of Environment, State Key Joint Laboratory of Environment Simulation and Pollution Control, Tsinghua University, Beijing 100084, China; ^6^State Environmental Protection Key Laboratory of Sources and Control of Air Pollution Complex, Beijing 100084, China; ^7^Geriatrics, China-Japan Friendship Hospital, 2 Yinghua Dongjie, Hepingli, Chaoyang District, Beijing 100029, China; ^8^Zhengzhou Hospital of Traditional Chinese Medicine, Zhengzhou City, Henan Province 450008, China

## Abstract

Atmospheric fine particulate matter 2.5 (PM 2.5) may carry many toxic substances on its surface and this may pose a public health threat. Epidemiological research indicates that cumulative ambient PM2.5 is correlated to morbidity and mortality due to pulmonary and cardiovascular diseases and cancer. Mitigating the toxic effects of PM2.5 is therefore highly desired. Bufei Huoxue (BFHX) capsules have been used in China to treat pulmonary heart disease (cor pulmonale). Thus, we assessed the effects of BFHX capsules on PM2.5-induced pulmonary inflammation and the underlying mechanisms of action. Using Polysearch and Cytoscape 3.2.1 software, pharmacological targets of BFHX capsules in atmospheric PM2.5-related respiratory disorders were predicted and found to be related to biological pathways of inflammation and immune function. In a mouse model of PM2.5-induced inflammation established with intranasal instillation of PM2.5 suspension, BFHX significantly reduced pathological response and inflammatory mediators including IL-4, IL-6, IL-10, IL-8, TNF-*α*, and IL-1*β*. BFHX also reduced keratinocyte growth factor (KGF), secretory immunoglobulin A (sIgA), and collagen fibers deposition in lung and improved lung function. Thus, BFHX reduced pathological responses induced by PM2.5, possibly via regulation of inflammatory mediators in mouse lungs.

## 1. Introduction

China has a substantial air pollution problem and of haze episodes correlate to atmospheric particulate matter abundance [1–3]. Fine particulate matter with nominal mean aerodynamic diameters of 2.5 *μ*m or less is defined as particulate matter 2.5 (PM2.5). Although PM2.5 particles are small and buoyant, they carry many toxic substances on their surface and they can “hang” in the atmosphere for some time and travel great distances [4]. Much epidemiological data indicate that cumulative ambient fine particulate matter correlates to morbidity of and mortality from pulmonary, cardiovascular diseases, and cancer [5–12], and these problems represent a significant social and economic burden.

Based on systems biology and pharmacology, network pharmacology leverages network analysis and screens nodes to identify proteins critical to a disease for drug design [13, 14]. Network pharmacology can be used to investigate synergism of multicomponent drugs to identify high efficacy and low toxicity agents with multiple targets [15, 16]. Chinese herbal formulas often have numerous targets and are comprised of many substances, so network pharmacology may be useful for studying traditional Chinese medicine [17, 18].

Bufei Huoxue (BFHX) capsules contain three common Chinese herbal products,* Astragalus*, radix paeoniae rubra, and* Psoralea corylifolia*, and have been approved by the China Food and Drug Administration (Number Z20030063) for the treatment of pulmonary heart disease. Clinical studies suggest that these compounds improve pulmonary ventilation function and reduce silicosis and lung inflammation [19–21]. Thus, we studied BFHX capsules and PM2.5-related respiratory disease using a network pharmacology platform. The effects of BFHX capsules on PM2.5-induced pulmonary inflammation were evaluated and the underlying mechanisms of action were investigated in mice.

## 2. Materials and Methods

### 2.1. PM2.5-Related Diseases

PM2.5-related diseases were identified using Polysearch System (http://wishart.biology.ualberta.ca/polysearch/index.htm), a web-based text-mining system for extracting relationships among human diseases, genes, drugs, and metabolism. PM2.5-related diseases were searched and extracted statements were manually screened. According to *Z*-values, the top 10 diseases were selected for further study.

### 2.2. Prediction of Disease Pathways and BFHX Targets

All target genes or proteins for each BFHX component (*Astragalus*, radix paeoniae rubra, and* Psoralea corylifolia*) were obtained using Polysearch and diseases were selected from predictions based on keywords. Targets were networked and visualized using Cytoscape 3.2.1 software. Drug- and disease-related targets were merged using Merge to screen key target proteins.

### 2.3. PM2.5 Mouse Model and Drug Intervention

#### 2.3.1. PM2.5 Sampling and Suspension Preparation

PM2.5 samples were collected from three sites in Beijing in the summer and winter seasons of 2011 and 2012. The three sites were a roadside site located 2 m from the curb of the North 4th Ring Road, an urban site on the campus of Tsinghua University, and a rural site situated in the countryside of Miyun. Sampling was conducted by Anderson particulate sampler HBL-GUV (Thermo Fisher, Waltham, MA) equipped with a PTFE membrane for two consecutive weeks [22]. At the end of sample collection, the PTFE membrane carrying PM2.5 was cut into 1 × 3 cm pieces and placed in ultrapure water. Particles were eluted three times under ultrasonic oscillation, 40 min each. Liquid containing the particulate matter was filtered through six layers of sterile gauze and centrifuged at 12,000 rpm at 4°C for 30 min. The lower layer of the suspension was collected and vacuum-freeze dried to yield PM2.5 particles which were then stored at −20°C. To make a stock solution, PM2.5 particles were weighed and suspended in saline at the desired concentrations after being ultrasonicated for 15 min and sterilized. Samples were stored at 4°C. Detailed methods can be found in related papers by us and other authors [23, 24].

#### 2.3.2. Drugs

BFHX capsules were provided by Lei Yun Shang Pharmaceutical Limited (Suzhou, Jiangsu, China). The dry powder was taken from the capsules and suspended in distilled water. The mixture was stored at 4°C.

#### 2.3.3. Animals

Adult female ICR mice (Beijing HFK Biotechnology Co., Ltd., China; 22–26 g), were housed in separate cages with food and water freely available under standard laboratory conditions of 22–28°C and relative humidity of 50–60% with a 12 h light/12 h dark cycle. Animal treatment and maintenance were performed in accordance with requirements of and the study protocol was approved by the institutional animal care and use committee at the China-Japan Friendship Hospital in Beijing (Permit #: 150202). All efforts were made to minimize suffering.

Sixty ICR female mice were randomized into 3 groups: normal controls, PM2.5 exposure, and BFHX intervention groups (*N* = 20/group). Controls were given an intranasal instillation of saline (20 *μ*l/mouse) on days 1, 8, 15, and 22. The PM2.5 exposure group was given an intranasal instillation of PM2.5 suspension (40 mg/kg, 20 *μ*l/mouse) on days 1, 8, 15, and 22 and each received oral saline (0.2 ml/mouse/day) from days 1 to 22. The BFHX intervention group was treated as was the PM2.5 exposure group except that BFHX (0.82 g/kg, 0.2 ml/mouse/day) instead of saline was given daily. Animals were sacrificed 48 h after the last treatment.

### 2.4. Lung Function

Mice were anesthetized with 2% pentobarbital (0.4 ml/40 g, ip) and secured in a supine position to be orotracheally intubated. Then, mice were transferred to a plethysmography platform and lung function analysis was measured lung include inspiratory resistance (RL), expiratory resistance (RE), dynamic lung compliance (Cdyn), and peak expiratory flow (PEF) using an AniRes2005 apparatus (Beilanbo Science and Technology Co., Ltd., Beijing, China).

### 2.5. Lung Histology

Mice were sacrificed and subjected to thoracotomy with lung resection. The left lung lobe was fixed in 10% formalin for 24 h and then dehydrated, embedded in paraffin and sectioned. Sections were stained with hematoxylin and eosin (H&E) according to the routine staining method and observed under an optical microscope. The right lung lobe was homogenized for molecular studies.

### 2.6. Masson Staining

Lung collagen content was quantified with Masson trichrome staining. Paraffin sections were first dewaxed, followed by washing with tap and then distilled water. Sections were then stained to identify nuclei with Regaud or Weigert hematoxylin for 5–10 min. After fully washing with water, slides were washed with distilled water and treated with Ponceau Masson Acid fuchsin solution for 5–10 min. Sections were then briefly immersed in a 2% aqueous acetic acid solution and then treated with 1% phosphomolybdic acid for 3–5 min, followed by direct staining with aniline blue or light green liquid dye for 5 min. After a brief Immersion in 0.2% acetic acid, sections were treated with 95% alcohol, ethanol, and transparent xylene and then cemented with neutral gum. For each slice, six fields (under 200x magnification) were selected. Positive Masson staining for collagen deposition was analyzed using by Image-Pro Plus multimedia color pathological image analysis software. The ratio of collagen deposition to the viewed area was calculated and averaged.

### 2.7. KGF and HMGB1 Expression in Lung Tissue

Keratinocyte growth factor (KGF) antibody was purchased from Biorbyt (Cambridge, UK), and the immunohistochemistry kit was from Gene Technology (Shanghai, China). High mobility group box 1 protein (HMGB1) antibody was purchased from Santa Cruz Biotechnology (Santa Cruz, CA), and the immunohistochemistry kit was from Gene Technology (Shanghai, China). Lung tissue sections were deparaffinized according to kit instructions and then stained with hematoxylin and viewed with light microscopy. Images were analyzed with Media Cybernetics Image-Pro Plus image analysis software. Each slice was assessed using 6 fields of integrated optical density (IOD) which represented protein expression.

### 2.8. Measurement of Inflammatory Mediators and sIgA by ELISA

The sacrificed mice were placed in a supine position and perfused with two 0.75 ml aliquots of ice-cold normal saline (NS). Each animal was lavaged five times. Lavage fluid was collected, and the cellular contents and bronchoalveolar lavage (BAL) fluid were separated by centrifugation. Supernatant was used to measure secretory immunoglobulin A (sIgA) with enzyme-linked immunosorbent assay (ELISA) kits (Abbexa, Cambridge, UK) according to the manufacturer's instructions. Optical density at 450 nm was measured with Microplate Reader 3 (MK3) (Lei Bo, Shanghai, China).

Lung tissue (0.1 g) was placed in a 1.5 ml EP tube to prepare 10% lung homogenate. Homogenate was centrifuged at 12,000 rpm and 4°C for 20 min. Supernatant used to measure inflammatory mediators from interleukin-4 (IL-4), interleukin-6 (IL-6), interleukin-10 (IL-10), interleukin-17 (IL-17), interleukin-1*β* (IL-1*β*), and tumor necrosis factor (TNF-*α*) with enzyme-linked immunosorbent assay (ELISA) kits (R & D, Minneapolis, MN) according to the manufacturer's instructions. Optical density at 450 nm was measured with Microplate Reader 3 (MK3) (Lei Bo, Shanghai, China).

### 2.9. Real-Time Reverse Transcriptase Polymerase Chain Reaction (RT-PCR)

RT-PCR was used to measure IL-8 and IgA expression in mouse lung tissues. Total RNA was extracted from 100 mg lung tissue with Trizol after a one-step extraction protocol. cDNA was synthesized by reverse transcription with 2 *μ*g RNA, 1 *μ*l oligo (dT), and DEPC water and 12 *μ*l lung tissue. *β*-Actin primer sequence is as follows: forward primer 5′-GTGACGTTGACATCCGTAAAGA-3′ and the reverse primer 5′-GTAACAGTCCGCCTAGAAGCAC-3′; IL-8 primer sequence is as follows: forward primer 5′-CATCTTCGTCCGTCCCTGTG-3′ and reverse primer 5′-GCCAACAGTAGCCTTCACCCA-3′; IgA primer sequence is as follows: forward primer 5′-GCTACAGTGTGTCCAGCGTCCT-3′ and reverse primer 5′-TGCCAGACTCAGGATGGGTAAC-3′. Quantitative analysis was performed using the 2^−ΔΔCt^ method.

## 3. Statistics

Data are represented as means ± standard deviation. Statistical analysis was conducted with SPSS 17.0 software. Comparisons among multiple groups were analyzed with one-way ANOVA with a Tukey-Kramer post hoc correction. Single comparisons were made with an unpaired two-tailed Student's *t*-test. Differences were considered statistically significant if *p* < 0.05.

## 4. Results

### 4.1. PM2.5-Related Disease Prioritization and Predicted Targets

**Figure 1:**
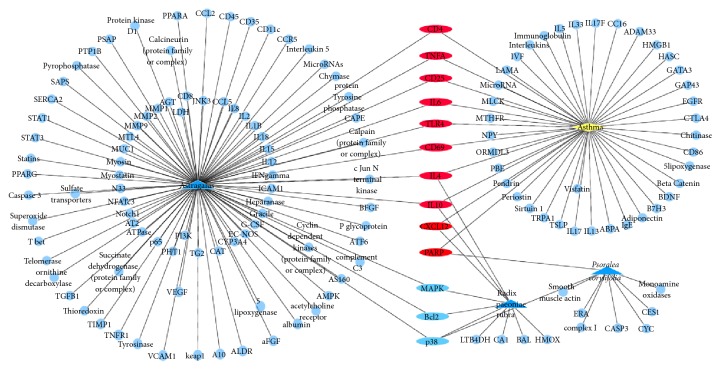
*Potential targets of BFHX in asthma*. Blue triangle represents* Astragalus*, radix paeoniae rubra, and* Psoralea corylifolia*; yellow diamond represents asthma; round blue nodes represent drug targets; red nodes represent drug targets of disease.

**Figure 2:**
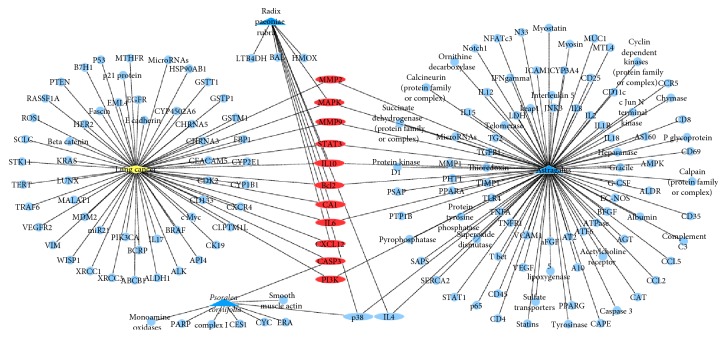
*Potential targets of BFHX in lung cancer*. Blue triangle represents* Astragalus*, radix paeoniae rubra, and* Psoralea corylifolia*; yellow diamond represents lung cancer; round blue nodes represent drug targets; red nodes represent drug targets of disease.

**Figure 3:**
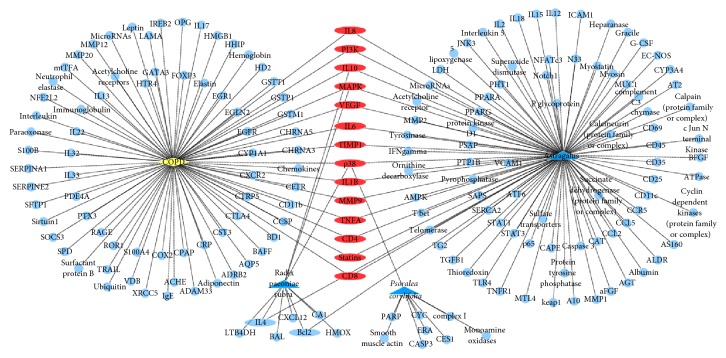
*Potential targets of BFHX in COPD*. Blue triangle represents* Astragalus*, radix paeoniae rubra, and* Psoralea corylifolia*; yellow diamond represents COPD; round blue nodes represent drug targets; red nodes represent drug targets of disease.

**Figure 4:**
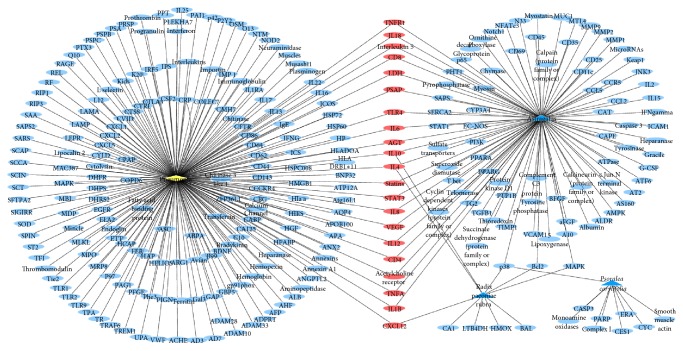
*Potential targets of BFHX in pneumonia*. Blue triangle represents* Astragalus*, radix paeoniae rubra, and* Psoralea corylifolia*; yellow diamond represents pneumonia; round blue nodes represent drug targets; red nodes represent drug targets of disease.

**Figure 5:**
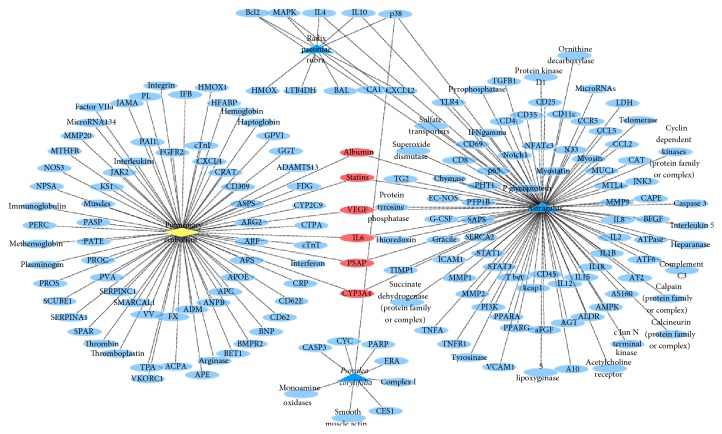
*Potential targets of BFHX in pulmonary embolism*. Blue triangle represents* Astragalus*, radix paeoniae rubra, and* Psoralea corylifolia*; yellow diamond represents pulmonary embolism; round blue nodes represent drug targets; red nodes represent drug targets of disease.

Polysearch platform analysis yielded a list of PM2.5 related diseases (Table 1), which were prioritized based on a relevancy score and expressed as a *Z* score, which refers to the number of standard deviations of the relevancy score above the mean value. A higher *Z* score denotes a lesser likelihood that the outcome is due to chance. Polysearch platform queries confirmed that 98, 10, and 9 target proteins were associated with* Astragalus*, radix paeoniae rubra, and* Psoralea corylifolia*, respectively (Figures 1–5).

### 4.2. Effect of BFHX on Mouse Pulmonary Function

**Figure 6:**
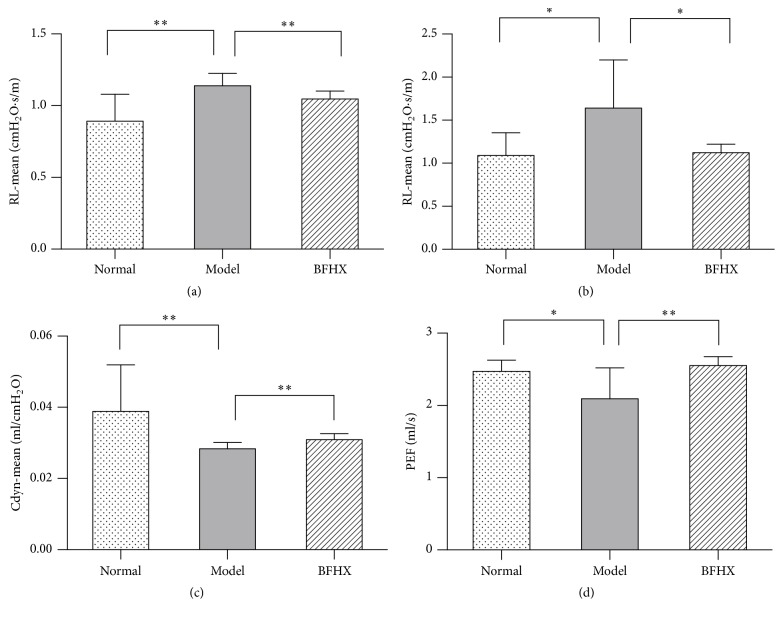
*Effect of BFHX on mouse pulmonary function*. Compared with PM2.5-exposed mice, the mean of lung inspiratory (a) and expiratory resistance (b) decreased significantly after BFHX intervention. Dynamic lung compliance (c) and peak expiratory flow rate (d) significantly improved. Inhale and exhale resistance, airway compliance, and peak expiratory flow data are means ± SD; ^*∗*^*p* < 0.05 and ^*∗∗*^*p* < 0.01 compared with the PM2.5 exposure group, *n* ≥ 8.

Compared with controls, lung inspiratory and expiratory resistances were significantly greater (*p* < 0.01, *p* < 0.05) in PM2.5 mice. Airway compliance and maximum forced expiratory flow were less in PM2.5 mice. BFHX intervention significantly decreased inspiratory (*p* < 0.01) and expiratory resistance (*p* < 0.05) and increased dynamic lung compliance and maximum forced expiratory flow (*p* < 0.01) in PM2.5 mice. Thus, BFHX intervention significantly improved lung function and reduced airway resistance and increased airway compliance (Figure 6) in PM2.5 mice.

### 4.3. Effect of BFHX on PM2.5-Induced Pulmonary Histopathology in Mice

**Figure 7:**
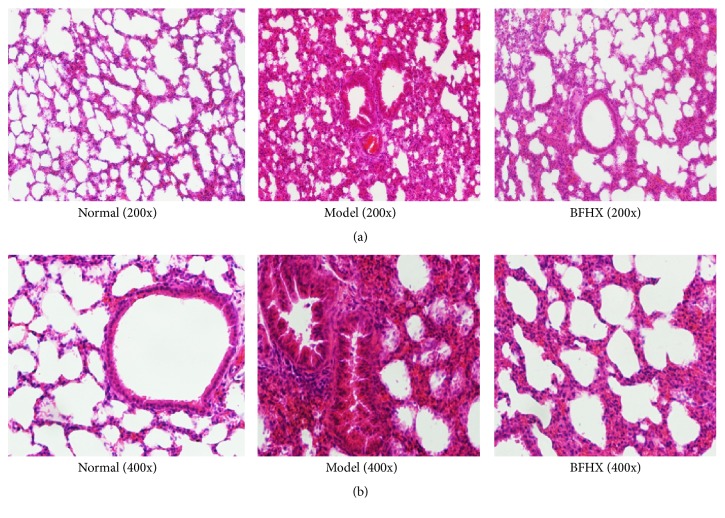
*Effect of BFHX on PM2.5-induced pulmonary histopathology in mice*. Pulmonary tissue of trachea and pulmonary interstitial inflammatory infiltration in PM2.5 model mice and BFHX groups. Inflammatory infiltration with BFHX was less compared with the PM2.5 exposure group. (a) 200x and (b) 400x.

In the normal group and the BFHX group, bronchial mucosa and bronchial wall structures were intact and alveolar structural integrity was intact. Some inflammatory cells had infiltrated alveolar spaces, but there was little interstitial inflammation and lung capillary structures were intact with no bleeding.  Figure 7 shows that compared with the normal group and the BFHX group, PM2.5 exposure caused histological injury such as widened alveolar septa, capillary congestion, and minor hemorrhages of small airways. BFHX treatment significantly reduced histological injury and reduced congestion and hemorrhage of small airways and recruitment.

### 4.4. Effect of BFHX on Lung Collagen Deposition in Mice

**Figure 8:**
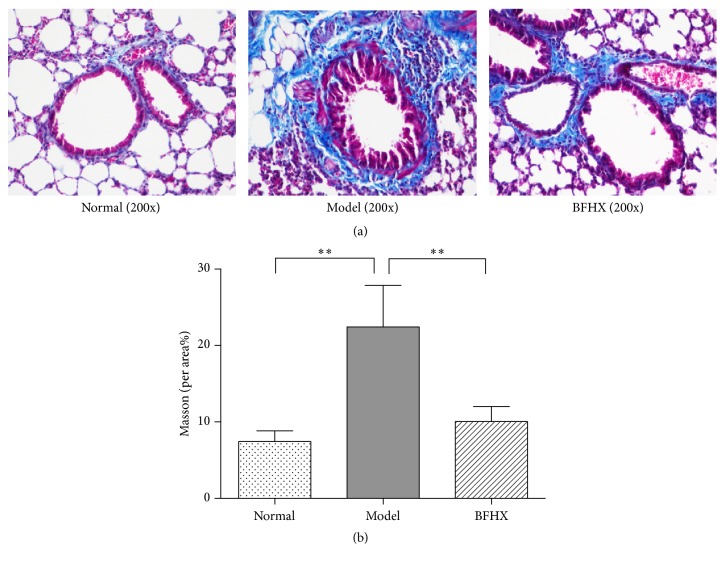
*Effect of BFHX on lung collagen deposition in mice*. (a) Light microscopy (200x). Blue represents collagen fibers and mucus; red represents cartilage, cytoplasm, muscle, cellulose, and glia and dark blue represents nuclei. (b) Percent of fibrotic lung tissue in the entire field. ^*∗∗*^*p* < 0.01 compared with tissue from the PM2.5 exposure group, *n* = 9.

Controls had collagen deposition in many small airways and fibrosis was noted around the vascular wall. PM2.5-exposed lung tissues had thickened airways with obvious collagen deposition, smaller alveoli with wider septa, and obvious fibrosis. Compared with PM2.5 lungs, BFHX-treated tissue had less tracheal and lung interstitial fibrosis (Figure 8). Thus, BFHX significantly alleviated collagen deposition in PM2.5 mice.

### 4.5. Effect of BFHX on HMGB1 and KGF Expression in Mouse Lung Tissues

**Figure 9:**
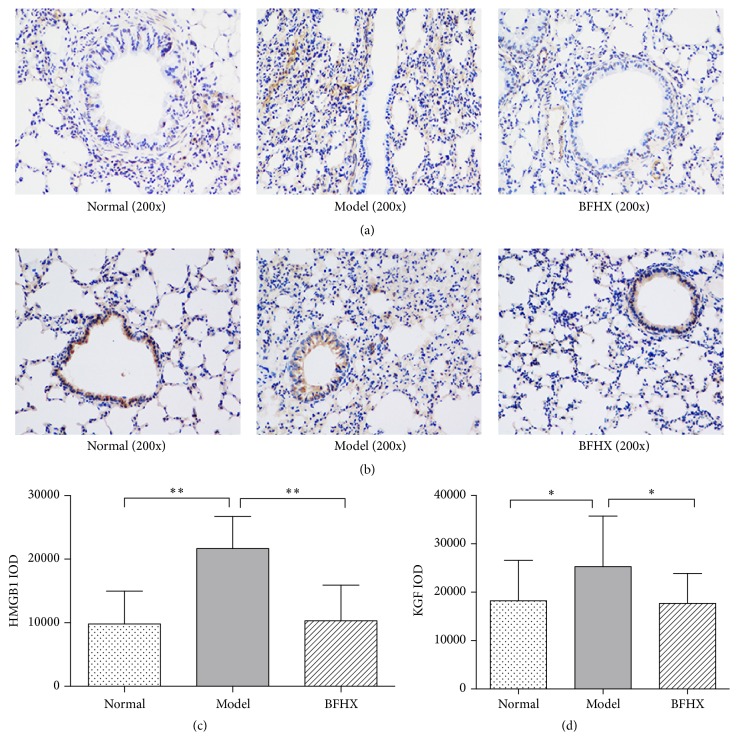
*Effect of BFHX on HMGB1 and KGF expression in PM2.5 mouse lung tissues*. (a) HMGB1 expression in alveolar tissues was significantly greater in PM2.5 tissues compared with controls and the BFHX intervention group (200x). (b) KGF expression in tracheal mucosa and alveolar tissues was significantly greater than in normal mice (200x). (c) HMGB1 expression quantified. (d) KGF expression quantified. Values are means ± SD; ^*∗*^*p* < 0.05, *n* = 9.

HMGB1 expression in alveolar tissue was significantly greater in PM2.5 tissues compared with controls (*p* < 0.01) and the BFHX intervention group (*p* < 0.01; Figures 9(a) and 9(c)), suggesting reduced inflammation was due to BFHX. Also, in lung tissue of PM2.5-exposed animals, KGF expression in tracheal mucosa and alveolar tissue was significantly greater than in normal mice and the BFHX intervention group (*p* < 0.05; Figures 9(b) and 9(d)).

### 4.6. Effect of BFHX on Inflammatory Mediator Expression in Mouse Lung Tissues

**Figure 10:**
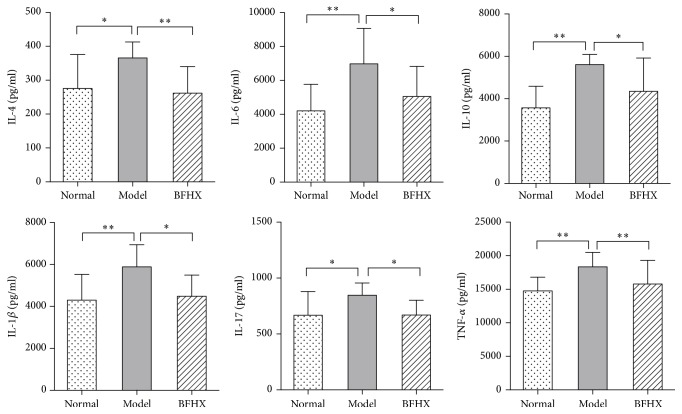
*Effect of BFHX on expression of inflammatory mediators*. BFHX-treated mice had less inflammation than untreated PM2.5-exposed mice. IL-4, IL-6, IL-10, IL-17, 1L-1*β*, and TNF-*α* expression in mice (means ± SD). ^*∗*^*p* < 0.05 and ^*∗∗*^*p* < 0.01 compared with the PM2.5 exposure group, *n* ≥ 6.

Compared with normal groups, IL-4, IL-6, IL-10, IL-1*β*, IL-17, and TNF-*α* was greater in lung tissue of PM2.5 model mice and BFHX groups. After treatment with BFHX, cytokines were reduced compared with model groups (Figure 10).

### 4.7. Effect of BFHX on IL-8, IgA, and sIgA Expression across Treatment Groups

**Figure 11:**
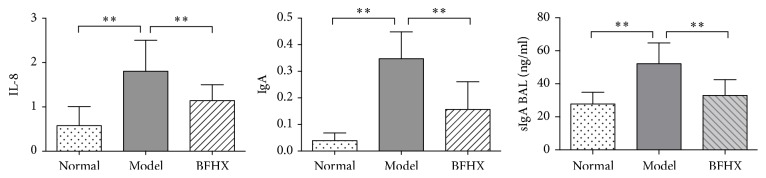
*IL-8, IgA, and sIgA expression in different mouse groups*. Control lung tissue homogenates had less IL-8 and IgA mRNA than PM2.5-exposed mouse tissue. sIgA expression in BAL fluid is less than PM2.5-exposed mouse BAL fluid; ^*∗∗*^*p* < 0.01, *n* ≥ 7.

Control lung tissue homogenates had less IL-8 and IgA mRNA than those measured in PM2.5-exposed mouse tissue (*p* < 0.01 for both). sIgA expression in the BAL fluid is less than that measured in PM2.5-exposed mouse BAL fluid (*p* < 0.01 for both). BFHX treatment reduced IL-8, IgA, and sIgA significantly (*p* < 0.01 for both) compared with mouse from the PM2.5 exposure group (Figure 11).

## 5. Discussion

Leveraging bioinformatics and computer science, network pharmacology offers an effective approach to studying drug and disease interactions by constructing a “molecular-target-disease” relationship [17]. Using a network pharmacology platform, we confirmed that PM2.5 exposure contributed to respiratory and heart disease. The respiratory system was initially injured but the circulatory and neural systems could be subsequently involved via multipathological pathways. Data show that BFHX targets many genes related to PM2.5 respiratory diseases, such as asthma, lung cancer, COPD, pneumonia, and pulmonary embolism in complicated and diverse ways. BFHX regulates expression of inflammatory factors (TNF-*α*, IL-1*β*, IL-4, IL-6, and IL-10) and immune regulation (CD4^+^ and CD8^+^ cells) and may be involved in adjusting related signaling pathways (BCL2, STAT3, and P38) in regulating cell proliferation, chemotaxis, and apoptosis (Figures 1–5).

Different sized particles can damage different parts of the respiratory system. Fine particulate matter (1–2.5 *μ*m diameter; PM1–2.5) can enter bronchi and the deeper respiratory tract. Those of 0.1–1 *μ*m diameter (PM0.1–1) can deeply penetrate the lung and ultrafine particles (PM0.1) can penetrate alveoli and enter the circulation [25]. PM2.5 can reduce lung function and induce asthma episodes, bronchitis, lung cancer, and other respiratory diseases [26]. Animal models of lung injury can be established by nasally instilling PM2.5 suspensions and in mice this manifests as lung tissue and small airway damage as well as alveolar and pulmonary interstitial inflammation. BFHX repaired airway damage and improved lung function in these models and decreased airway resistance and increased lung compliance. Data show that BFHX can improve lung function reducing PM2.5-induced lung injury and promoting healing. BFHX also significantly reduced collagen deposition which may improve fibrosis and improve lung function (Figure 6).

TNF-*α* and IL-1*β* are important proinflammatory cytokines that activate and recruit inflammatory cells, enhance inflammatory response [27, 28], and stimulate proliferative response of smooth muscle cells and fibroblast in the airway, resulting in remodeling [29, 30]. IL-4 is a growth factor secreted by T cells and IL-6, produced by responses of mononuclear macrophages, endothelial cells, fibroblasts, and other cells to IL-1 and TNF-*α* [31–33], contributes to immune defenses and can enhance airway inflammation. IL-10 is an immunomodulatory cytokine and is made mainly by activated monocytes, lymphocytes, and epithelial cells. A high level of IL-10 was produced after damage [34, 35] and IL-8 was significantly increased in local inflammation, serum and body fluid because of infection, and some autoimmune diseases [36, 37]. BFHX significantly decreased TNF-*α*, IL-1*β*, IL-6, IL-10, IL-4, and IL-8 in lung of PM2.5 mode mice (Figures 10 and 11).

HMGB1 is widely distributed in the lymph, brain, liver, lung, heart, kidney, and other tissues. Under stimulation of IL-1 and TNF-*α*, HMGB1 can be secreted into the extracellular space to promote cytokine production and extend inflammation [38]. Recent studies indicate that activation of HMGB1 signaling is associated with acute lung injury, asthma, pulmonary fibrosis, and other respiratory diseases and HMGB1 expression is correlated with respiratory disease severity [39]. BFHX alleviated inflammation and decreased subsequent injury to lung tissue of PM2.5 model mice (Figure 9).

KGF is a cytokine synthesized by *γδ*T cells and stimulates epithelial cell proliferation, differentiation, and migration and promotes mucosal repair maintenance of mucosal integrity. KGF stimulates synthesis, storage, and secretion of pulmonary surfactant in type II alveolar epithelial cells (AEC II), reduces alveolar surface gas-liquid surface tension, and prevents excessive expiratory alveolar collapse and over-inspiratory expansion to maintain alveolar shape. KGF secretion is increased, promoting tissue repair, but KGF is reported to be associated with early growth of mesothelial cells after asbestos exposure, which contributes to lung fibrosis [40]. IL-17, a powerful proinflammatory factor, is a major cytokines synthesized by *γδ*T cells and reflects their biological activity to a certain extent [41]. *γδ*T cells are important during the transition from innate immune cells to acquired immune cells. So BFHX may control *γδ*T cell activity to reduce lung injury of in PM2.5 model mice and reduce expression of KGF to reduce PM2.5-induced lung fibrosis (Figures 9 and 10).

sIgA is a human IgA exocrine antibody found in the mucosa of respiratory, gastrointestinal, and urogenital tracts and is a first line of defense against bacterial and viral adsorption and colonization on epithelial cell surfaces [6]. Respiratory tract infections, pathogenic microbial invasion, and adhesion to the epithelium stimulate a local mucosal immune response by increasing synthesis and secretion of sIgA. Data show that in PM2.5 mice, BFHX reduced sIgA secretion and reduced airway damage, suggesting that BFHX ameliorated local mucosal inflammation (Figure 11). In summary, our data suggest that BFHX reduced pathological responses induced by PM2.5 via regulation of inflammatory mediators in mouse lungs.

## 6. Conclusion

We noted that PM2.5 could stimulate lung tissue inflammatory factors, promote generation of secretory immunoglobulin and KGF, and cause collagen fiber deposition in lung tissue. BFHX modified the respiratory tract mucosal immune response and inhibited secretion of immunoglobulin to reduce release of inflammatory cytokines and collagen fiber deposition. It also improved lung function and alleviated injury in a PM2.5 mouse model.

## Figures and Tables

**Table 1:** PM2.5-related diseases identified through Polysearch.

Number	*Z* score	Disease name
1	9	Respiratory disease
2	8.1	Asthma
3	7.7	Lung cancer
4	5.8	Cardiovascular disease
5	3.7	COPD
6	2.2	Atherosclerosis
7	1.2	Ischemic stroke
8	0.8	Pneumonia
9	0.7	Gestational diabetes mellitus
10	0.7	Pulmonary embolism

## References

[1] Zhao Q., He K.-B., Ma Y.-L. (2009). Regional PM pollution in Beijing and surrounding area during summertime. *Huan Jing Ke Xue*.

[2] Watson J. G. (2002). Visibility: science and Regulation. *Journal of the Air and Waste Management Association*.

[3] Wang S., Hao J. (2012). Air quality management in China: issues, challenges, and options. *Journal of Environmental Sciences*.

[4] Mu Z., Zhao J., Xu N., Yao L., Li Y., Meng J. (2011). Vertical and temporal variation of PM10 in Yanta District, Xi'an during winter. *Acta Scientiae Circumstantiae*.

[5] Pope C. A., Burnett R. T., Thun M. J. (2002). Lung cancer, cardiopulmonary mortality, and long-term exposure to fine particulate air pollution. *The Journal of the American Medical Association*.

[6] Ma Y., Chen R., Pan G. (2011). Fine particulate air pollution and daily mortality in Shenyang, China. *Science of the Total Environment*.

[7] Li N., Peng X., Zhang B., Yin W., Yu S. (2010). A time-series study between air pollutant and the respiratory daily mortality in Guangzhou. *Journal of Huazhong University of Science and Technology. Health Sciences*.

[8] Guo Y., Barnett A. G., Zhang Y., Tong S., Yu W., Pan X. (2010). The short-term effect of air pollution on cardiovascular mortality in Tianjin, China: comparison of time series and case-crossover analyses. *Science of the Total Environment*.

[9] Zhang Y.-S., Zhou M.-G., Jia Y.-P. (2010). Time-series analysis of association between inhalable particulate matter and daily mortality among urban residents in Tianjin. *Zhonghua Liu Xing Bing Xue Za Zhi*.

[10] Orru H., Teinemaa E., Lai T. (2009). Health impact assessment of particulate pollution in Tallinn using fine spatial resolution and modeling techniques. *Environmental Health: A Global Access Science Source*.

[11] Ren Y.-J., Li X.-Y., Jin M.-J., Chen K., Xiang H.-Q., Liu Q.-M. (2007). Case-crossover studies of air particulate matter pollution and cardiovascular disease death. *China Environmental Science*.

[12] Zhang J., Meng H., Zhang G., Zhang Z., Zhao W., Pan X. (2011). Relationship between air pollution and daily respiratory system disease mortality in Chaoyang District, Beijing: a time-series analysis. *Journal of Environmental and Health*.

[13] Hsin K.-Y., Ghosh S., Kitano H. (2013). Combining machine learning systems and multiple docking simulation packages to improve docking prediction reliability for network pharmacology. *PLoS ONE*.

[14] Tao W., Xu X., Wang X. (2013). Network pharmacology-based prediction of the active ingredients and potential targets of Chinese herbal Radix Curcumae formula for application to cardiovascular disease. *Journal of Ethnopharmacology*.

[15] Wang Y., Gao X., Zhang B., Cheng Y. (2011). Building methodology for discovering and developing Chinese medicine based on network biology. *Zhongguo Zhong Yao Za Zhi*.

[16] Prado-Prado F. J., Uriarte E., Borges F., González-Díaz H. (2009). Multi-target spectral moments for QSAR and complex networks study of antibacterial drugs. *European Journal of Medicinal Chemistry*.

[17] Azmi A. S., Mohammad R. M. (2014). Rectifying cancer drug discovery through network pharmacology. *Future Medicinal Chemistry*.

[18] Cheng B.-F., Hou Y.-Y., Jiang M., Zhao Z.-Y., Dong L.-Y., Bai G. (2013). Anti-inflammatory mechanism of Qingfei Xiaoyan Wan studied with network pharmacology. *Yaoxue Xuebao*.

[19] Wu D., Xie Z., Di P. (2012). Clinical effect of BuFeiHuoXue capsules. *China Practical Medicine*.

[20] Li B., Su L. (2006). Clinical observation of BuFeiHuoXue capsules effect on lung ventilation. *Practical Journal of Cardiac Cerebral Pneumal and Vascular Disease*.

[21] Tian L., Cao G., Liu G. (2014). Effect of BuFeiHuoXue capsules on pneumosilicosis. *Guangdong Yi Xue*.

[22] Wu Y., Yang L., Zheng X. (2014). Characterization and source apportionment of particulate PAHs in the roadside environment in Beijing. *Science of the Total Environment*.

[23] Qin Y., Jing Y., Liu Y. (2016). Influence of Guben Zhike Granules on lung function and morphology of lung injury mouse model induced by PM2.5. *China Journal of Traditional Chinese Medicine and Pharmacy*.

[24] Jiang Z.-H., Song W.-M. (2005). Effects of NF-*κ*B in acute lung injury induced by PM2.5 in mice. *Journal of Environmental and Occupational Medicine*.

[25] Yang H., Zou X., Wang H., Liu Y. (2012). Study progress on PM2.5 in atmospheric environment. *Journal of Meteorology and Environment*.

[26] Yang X., Feng L., Wei P. (2012). Atmospherical PM2.5 and its harmness. *Frontier Science*.

[27] Bhatia M., Moochhala S. (2004). Role of inflammatory mediators in the pathophysiology of acute respiratory distress syndrome. *Journal of Pathology*.

[28] Ming W. J., Bersani L., Mantovani A. (1987). Tumor necrosis factor is chemotactic for monocytes and polymorphonuclear leukocytes. *Journal of Immunology*.

[29] Ma R., Chen J., Li Z. Y., Tang J. C., Wang Y. F., Cai X. J. (2014). Decorin accelerates the liver regeneration after partial hepatectomy in fibrotic mice. *Chinese Medical Journal*.

[30] Bossa A. S., Salemi V. M. C., Ribeiro S. P. (2014). Plasma cytokine profile in tropical endomyocardial fibrosis: predominance of TNF-a, IL-4 and IL-10. *PLoS ONE*.

[31] Lin C.-Y., Zhang H., Cheng K.-C., Slutsky A. S. (2003). Mechanical ventilation may increase susceptibility to the development of bacteremia. *Critical Care Medicine*.

[32] Jones S. A., Richards P. J., Scheller J., Rose-John S. (2005). IL-6 transsignaling: the in vivo consequences. *Journal of Interferon and Cytokine Research*.

[33] Mora A. L., Torres-González E., Rojas M. (2006). Activation of alveolar macrophages via the alternative pathway in herpesvirus-induced lung fibrosis. *American Journal of Respiratory Cell and Molecular Biology*.

[34] Kabyemela E. R., Muehlenbachs A., Fried M., Kurtis J. D., Mutabingwa T. K., Duffy P. E. (2008). Maternal peripheral blood level of IL-10 as a marker for inflammatory placental malaria. *Malaria Journal*.

[35] Liu J., Huang S., Su X.-Z., Song J., Lu F. (2016). Blockage of Galectin-receptor interactions by *α*-lactose exacerbates plasmodium berghei-induced pulmonary immunopathology. *Scientific Reports*.

[36] Giebelen I. A. J., Van Westerloo D. J., LaRosa G. J., De Vos A. F., Van Der Poll T. (2007). Local stimulation of *α*7 cholinergic receptors inhibits LPS-induced TNF-*α* release in the mouse lung. *Shock*.

[37] Nakayama S., Mukae H., Ishii H. (2005). Comparison of BALF concentrations of ENA-78 and IP10 in patients with idiopathic pulmonary fibrosis and nonspecific interstitial pneumonia. *Respiratory Medicine*.

[38] Huang W., Tang Y., Li L. (2010). HMGB1, a potent proinflammatory cytokine in sepsis. *Cytokine*.

[39] Gangemi S., Casciaro M., Trapani G. (2015). Association between HMGB1 and COPD: a systematic review. *Mediators of Inflammation*.

[40] Adamson I. Y. (1997). Early mesothelial cell proliferation after asbestos exposure: in vivo and in vitro studies. *Environmental Health Perspectives*.

[41] Zhang G., Zhou K. F., Lu Z. H. (2016). Interleukin-17 enhances the removal of respiratory syncytial virus in mice by promoting neutrophil migration and reducing interferon-gamma expression. *Genetics and Molecular Research*.

